# F-Type ATP Synthase Assembly Factors Atp11 and Atp12 in Arabidopsis

**DOI:** 10.3389/fpls.2020.522753

**Published:** 2020-10-19

**Authors:** Zhikun Duan, Kaiwen Li, Lin Zhang, Liping Che, Lizhen Lu, Jean-David Rochaix, Congming Lu, Lianwei Peng

**Affiliations:** ^1^State Key Laboratory of Crop Stress Adaptation and Improvement, School of Life Sciences, Henan University, Kaifeng, China; ^2^Shanghai Key Laboratory of Plant Molecular Sciences, College of Life Sciences, Shanghai Normal University, Shanghai, China; ^3^Key Laboratory of Photobiology, Institute of Botany, Chinese Academy of Sciences, Beijing, China; ^4^Departments of Molecular Biology and Plant Biology, University of Geneva, Geneva, Switzerland; ^5^State Key Laboratory of Crop Biology, College of Life Sciences, Shandong Agricultural University, Tai’an, China

**Keywords:** ATP synthase, assembly, chloroplast, mitochondria, Atp11, Atp12

## Abstract

Atp11p and Atp12p are members of two chaperone families essential for assembly of the mitochondrial ATP synthase in *Saccharomyces cerevisiae* and *Homo sapiens*. However, the role of their homologs in higher plants is unclear with regard to the assembly of both chloroplast ATP synthase (cpATPase) and mitochondrial ATP synthase (mtATPase). Here, we show that loss of either Atp11 or Atp12 is lethal in Arabidopsis. While Atp12 is only localized in mitochondria, Atp11 is present both in chloroplasts and mitochondria. Yeast two-hybrid analyses showed that, as their homologs in yeast, Atp11 specifically interacts with the β subunit of the mtATPase and cpATPase, and Atp12 interacts with the α subunit of the mtATPase, implying that Atp11 and Atp12 fulfill a conserved task during assembly of ATP synthase. However, the binding sites for Atp11 in the β subunit of mtATPase and cpATPase are slightly different, suggesting that the mechanisms of action may have evolved in different ways. Although Atp11 interacts with cpATPase β subunit as the two assembly factors BFA3 and BFA1, they bind to different sites of the β subunit. These results indicate that Atp11 is involved in the assembly of both cpATPase and mtATPase but Atp12 is specifically required for the assembly of mtATPase in higher plants.

## Introduction

Adenosine triphosphate (ATP) is the major cellular energy storage compound used to drive numerous biochemical reactions in biological systems. Utilizing the proton motive force (pmf) across the membrane, ATP is synthesized by the ubiquitous membrane-embedded F_1_F_*o*_ ATP synthase, also called F-type ATP synthase (Mitchell, 1961; Yoshida et al., 2001; Junge and Nelson, 2015). F-type ATP synthase is a multiprotein complex widely distributed in bacteria, mitochondria (mtATPase) and chloroplasts (cpATPase) (Bertoni, 2018). These enzymes share a similar structure composed of two rotary motors with distinct functionalities. The F_1_ subcomplex represents the hydrophilic motor which protrudes from the thylakoid membrane into the stroma and contains five subunits α, β, γ, δ, and ε, with a stoichiometric ratio of 3:3:1:1:1 (von Ballmoos et al., 2009; Hahn et al., 2018). Three catalytic nucleotide and phosphate binding sites are located at the interfaces between the α and β subunits. The α_3_β_3_ hexamer surrounds a central stalk containing the subunits γ and ε (Abrahams et al., 1994; Groth and Pohl, 2001). The F_*o*_ motor is a hydrophobic membrane-integrated component with the subunit composition *ab*_2_*c*_10__–__15_ (Nakamoto et al., 2008; von Ballmoos et al., 2009). F_1_ is fixed and physically connected to F_*o*_ via the subunits *b*_2_ in F_*o*_ and δ in F_1_. The γ/ε stalk is inserted into the α_3_β_3_ hexamer and fixed on the *c*-ring of F_*o*_. During ATP synthesis, the transmembrane electrochemical proton gradient generated by photosynthetic or mitochondrial electron transport drives the rotation of the *c*-ring and the γ/ε stalk, thereby inducing conformational changes in the catalytic sites of the α_3_β_3_ hexamer that lead to synthesis of ATP from ADP and inorganic phosphate (Junge and Nelson, 2015; Kühlbrandt, 2019).

Although the structure of the F-type ATP synthase and the functional mechanisms of ATP synthesis have been widely investigated, the assembly of the complex is not well understood. Previous studies revealed that assembly of both mtATPase and cpATPase proceeds in a stepwise manner (Ackerman and Tzagoloff, 2005; Rak et al., 2011; Rühle and Leister, 2015; Zhang et al., 2020). During their assembly, several assembly factors are required at certain steps through specific interactions with individual subunits of the ATP synthase. Atp11p and Atp12p are two assembly factors present in most eukaryotes and they are essential for assembly of mtATPase (Ackerman and Tzagoloff, 2005; Pícková et al., 2005; Rak et al., 2011). In *Saccharomyces cerevisiae*, dysfunction of the Atp11p and Atp12p proteins induces aggregation of the F_1_β and F_1_α subunits, suggesting that Atp11p and Atp12p are required to maintain the β and α subunits in a soluble state at the early stage of ATP synthase assembly (Wang and Ackerman, 2000; Wang et al., 2000). Further investigations revealed that Atp11p binds to the pre-catalytic site (CS) at the surface of unassembled β subunit while Atp12p binds to the pre-noncatalytic site (NCS) interface at the surface of the unassembled α subunit to form Atp11p-β and Atp12p-α heterodimers, respectively (Wang and Ackerman, 2000; Wang et al., 2000; Ackerman and Tzagoloff, 2005). This kind of interaction is believed to prevent the formation of insoluble α–α and β–β homodimers (Wang et al., 2000). Structures of Atp11p from *Candida glabrata* and Atp12p from *Paracoccus denitrificans* have been resolved and it has been proposed that formation of Atp11p-β and Atp12p-α heterodimers may act as decoys preventing the formation of nonphysiological α/β subunit aggregates (Ludlam et al., 2009).

Homologs of Atp11p and Atp12p are also present in higher plants that contain two F-type ATP synthases, cpATPase and mtATPase. We designated them Atp11 and Atp12, respectively. However, their localization and functions in higher plants remain unclear. In this study, we provide evidence that absence of Atp11 or Atp12 is lethal for Arabidopsis. While Atp12 is localized in mitochondria and interacts with the α subunit of mtATPase, Atp11p is localized in both chloroplasts and mitochondria. Both proteins may fulfill similar functions as their orthologs during the assembly of F-type ATP synthase in higher plants.

## Results

### Isolation and Characterization of the *atp11* and *atp12* Mutants

**FIGURE 1:**
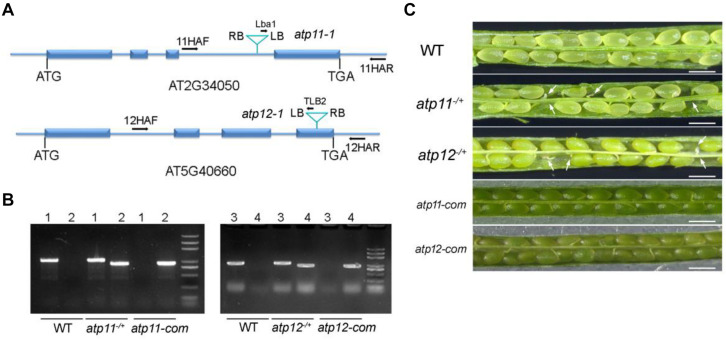
Phenotype of the *atp11* and *atp12* mutants. **(A)** Schematic diagram of the *Atp11* and *Atp12* genes and the positions of the T-DNA insertions are indicated. Blue boxes represent exons and blue lines represent introns or parts of UTRs. **(B)** PCR analysis of genomic DNA from the WT, *atp11*^–^*^/+^*, *atp12*^–^*^/+^* and their complementation lines. Forward (11HAF or 12HAF), reverse primers (11HAR or 12HAR) as well as Lba1 and TLB2 primers are indicated in Figure 1A, which were used to confirm the heterozygosity of the mutants and the insertions of the T-DNA in the *atp11-1* and *atp12-1* mutants. 1, 11HAF+11HAR; 2, Lba1+11HAR; 3, 12HAF+12HAR; 4, TLB2+12HAF. **(C)** Siliques of WT, *atp11-1* and *atp12-1* heterozygous after flowering were photographed by light microscopy. *atp11*-com and *atp12*-com, complemented lines for *atp11-1* and *atp12-1*, respectively. Bars = 0.5 mm.

To investigate the roles of Atp11 and Atp12 in Arabidopsis, the T-DNA insertion mutants *atp11-1* (SALK_018817) and *atp12-1* (GABI_516A01) were obtained from the Arabidopsis biological resource center (ABRC). To determine the T-DNA insertion sites, genomic DNA was extracted from the mutants and the amplified products were subsequently sequenced (Figures 1A,B). The results showed that the T-DNA was inserted into the third intron and fourth exon of *Atp11* and *Atp12* in the corresponding mutants, respectively (Figure 1A). However, neither *atp11-1* nor *atp12-1* homozygous mutants could be identified (Figure 1B). No visible phenotype was found in the *atp11-1* or *atp12-1* heterozygous mutants. Failure to obtain homozygous mutant plants suggested homozygous lethality for these two genes. We then analyzed the seeds within mature siliques of the heterozygous mutants. Both mutants display a deficiency in endosperm or embryo development (Figure 1C). We then determined the ratio of normal to aborted embryos in the progeny of *atp11* and *atp12* heterozygous plants. The results showed that in both cases, this ratio is about 3:1 (Supplementary Table S1). Moreover, the ratio of wild type to heterozygous plants in the progeny of both *atp11* and *atp12* heterozygous is close to 1:2 (Supplementary Table S2). These results indicate that the *atp11* and *atp12* mutants and their corresponding T-DNA insertion are inherited in a Mendelian manner.

Full-length genomic gene sequences including their promoters were used to complement the *atp11-1* and *atp12-1* heterozygous mutants. The complemented transgenic lines in the *atp11-1* and *atp12-1* homozygous mutant background were screened in the second generation by PCR (Figure 1B). As shown in Figure 1C, the complemented plants produced fully developed and viable seeds similar to WT (Figure 1C), confirming that the embryo lethality of the *atp11-1* and *atp12-1* homozygous mutants was indeed due to the disruptions of *Atp11 and Atp12*, respectively.

### Sequence Alignment of Atp11 and Atp12 Proteins From Eukaryotic Photosynthetic Organisms

**FIGURE 2:**
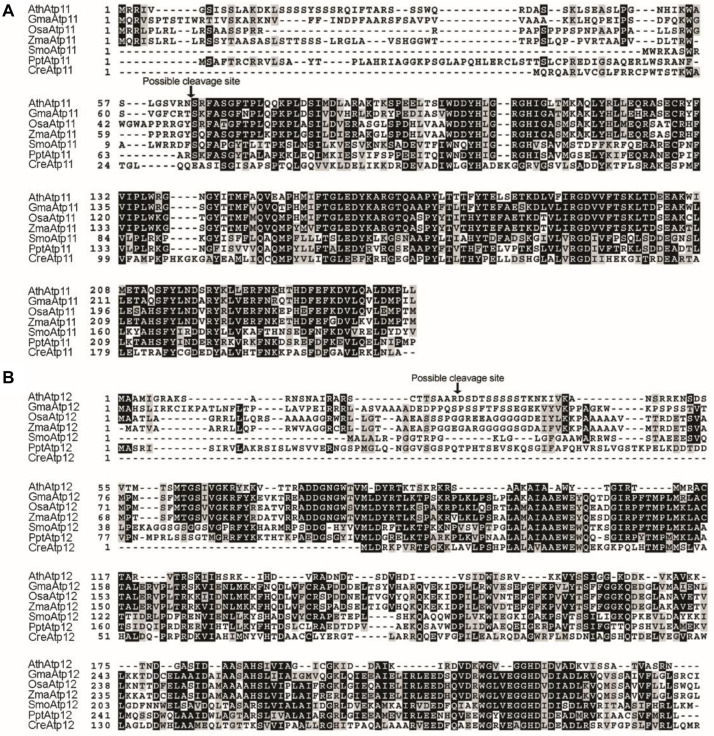
Sequence alignment of Atp11 and Atp12 proteins from eukaryotic photosynthetic organisms. **(A)** Multiple sequence alignment of Atp11 and several orthologs. **(B)** Multiple sequence alignment of Atp12 and several orthologs. Sequences of Atp11 and Atp12 were obtained from GenBank (http://www.ncbi.nlm.nih.gov/) or Phytozome (http://www.phytozome.net/) and aligned with ClustalW2. The possible cleavage site of Atp11 and Atp12 from Arabidopsis were predicted with the CBS Prediction Servers (http://www.cbs.dtu.dk/services) and are indicated by a black arrow above the sequence. Identical amino acids are shown with white letters on a black background, and similar amino acids are indicated with black letters on a gray background.

Bioinformatic analysis indicates that there are proteins related to Atp11 and Atp12 in various land plants as well as in the green alga *Chlamydomonas reinhardtii* (Figure 2), but not in cyanobacteria. Multiple sequence alignment analysis showed that the sequences of Atp11 and Atp12 are well conserved (Figure 2). Besides, Atp11 and Atp12 are predicted to have a plastid- and mitochondrial-targeting peptide, respectively, at their N termini by ChloroP analysis^1^ (Figure 2).

### Subcellular Localization of Atp11 and Atp12 Proteins in Arabidopsis

**FIGURE 3:**
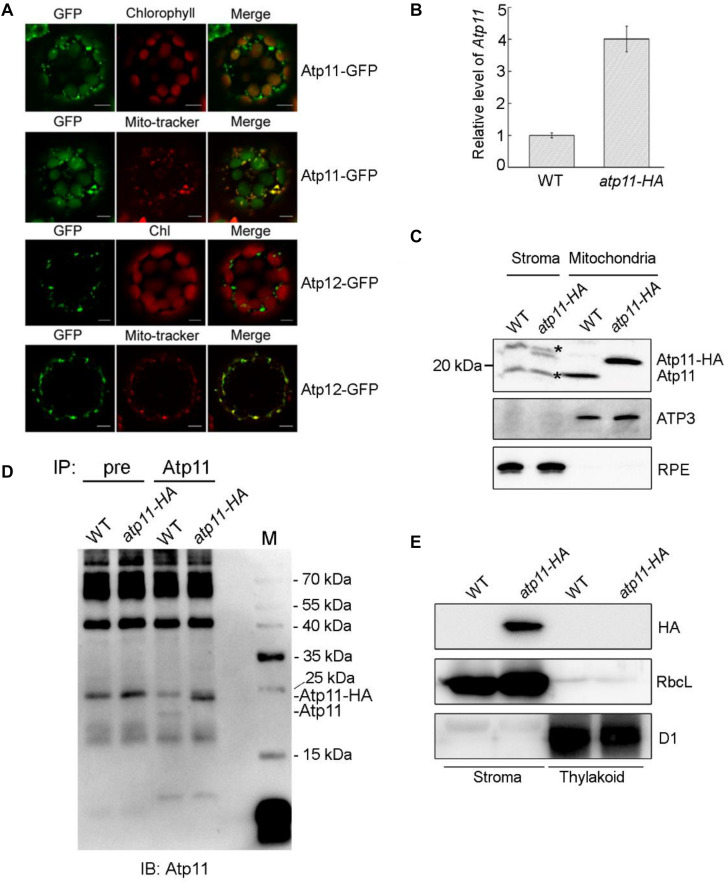
Subcellular localization of Atp11 and Atp12 proteins in plants. **(A)** Subcellular localization of Atp11 and Atp12 in Arabidopsis protoplasts. Atp11-GFP, Atp11-GFP fusion; Atp12-GFP, Atp12-GFP fusion; Mito-tracker, Mito-tracker Red. Bars = 20 μm. **(B)** Relative expression of *Atp11* transcripts in WT and *atp11-HA* transgenic plants. Three-week-old plants were used for quantitative RT-PCR. *Actin 2* gene was used as control. Error bars indicate ± SD (*n* = 3). **(C)** Immunoblot analysis of Atp11 in chloroplast stroma and mitochondria. A total of 80 μg chloroplast stroma protein and 20 μg mitochondrial protein was loaded on each lane for detection of Atp11. Antibodies against mitochondrial protein ATP3 and chloroplast protein RPE (D-RIBULOSE-5-PHOSPHATE-3-EPIMERASE) were used as loading and fractionation controls. Black asterisks indicate non-specific bands. **(D)** Immunoprecipitation of chloroplast stroma-localized Atp11. Chloroplast stroma extracted from wild type and *atp11-HA* plants were incubated with CNBr-activated agarose coupled with preimmune serum (pre) and Atp11 antibody. The bound proteins were eluted and detected with Atp11 antibody. **(E)** Immunoblot analysis of Atp11 in chloroplasts. Intact chloroplasts were isolated from wild type and *atp11-HA* and separated into stroma and thylakoid membrane fractions. Immunoblot analysis was performed with HA antibody. D1 and RbcL were used as loading and fractionation controls.

According to the prediction, Arabidopsis Atp11 has a chloroplast transit peptide at its N-terminal end, suggesting that it is localized in the chloroplast. In contrast, Arabidopsis Atp12 is predicted to localize in the mitochondria. To confirm their localization in plant cells, the coding regions of *Atp11* and *Atp12* were fused with GFP at their C-termini. The fusion proteins were expressed in Arabidopsis protoplasts (Figure 3A). It is clearly observed that Atp11-GFP fluorescence co-localizes with both chloroplasts and mito-tracker red (Figure 3A), indicating that Atp11 is targeted to both chloroplasts and mitochondria. In contrast, Atp12-GFP fluorescence exclusively overlaps with mito-tracker red (Figure 3A), indicating that Atp12 is localized in mitochondria.

To further determine the localization of Atp11 in Arabidopsis, we introduced the genomic *Atp11* gene fused with a sequence encoding the HA tag into the *atp11-1* heterozygous mutant. Transgenic lines in the *atp11-1* homozygous mutant background were screened by PCR and one of these transgenic lines named *atp11-HA* was chosen for further study. Quantitative RT-PCR (qRT-PCR) analysis showed that, although expression of *Atp11-HA* is driven by the authentic promoter of *Atp11*, the level of *Atp11-HA* transcript in the transgenic plants is about four times higher than the transcript level of *Atp11* in WT plants (Figure 3B). Chloroplast stroma and intact mitochondria isolated from WT and *atp11-HA* plants were used for immunoblot analyses using the antibody against Atp11. A protein with a molecular mass of less than 20 kDa was detected in the mitochondria of WT plants and this band is shifted to a higher molecular mass in the *atp11*-HA plants, as expected for the Atp11-HA fusion protein (Figure 3C). Comparable levels of ATP3 were detected in the mitochondria of WT and *atp11-HA* plants (Figure 3C). These results indicate that the Atp11 protein is localized in the mitochondria and that the addition of the HA-tag fused to the C-terminus of Atp11 does not affect the function of Atp11 during assembly of mtATPase.

The Atp11-HA fusion protein was also detected in the chloroplast stroma fraction of *atp11*-HA plants (Figure 3C). However, our antibody detected a non-specific band with a molecular mass similar to Atp11 protein in the chloroplast stroma (Figure 3C). To further confirm the presence of Atp11 in the chloroplast stroma of wild-type plants, immunoprecipitation experiments were performed. Chloroplast stroma from WT and *atp11*-HA plants was incubated with CNBr-activated agarose coupled with preimmune serum and Atp11 antibody overnight. Immunoblot analyses of the immunoprecipitated samples using the Atp11 antibody detected the Atp11 and Atp11-HA bands form WT and *atp11*-HA stroma, respectively (Figure 3D), indicating that Atp11 is indeed present in the stroma of chloroplasts. The level of Atp11-HA in the *atp11*-HA plants is higher than the level of Atp11 in the WT plants in both the chloroplast stroma and mitochondria (Figures 3C,D), which is consistent with the qRT-PCR results of *Atp11* transcripts in both genotypes (Figure 3B).

Intact chloroplasts isolated from *atp11*-HA plants were separated into stroma and thylakoid membrane fractions, which were further used for immunoblot analyses using the HA antibody. The Atp11-HA fusion protein was detected in the stroma fraction of *atp11*-HA plants, but no signal was detected in the stroma fraction of WT or thylakoid membranes of WT and *atp11*-HA plants (Figure 3E), indicating that Atp11 is not localized to the chloroplast thylakoids. Localization of Atp11 in chloroplast stroma is consistent with the localization of its ortholog in yeast as well as with the observation that no transmembrane domain is predicted in Atp11 (TMHMM program^2^).

Taken together, we conclude that while Atp12 is specifically targeted to the mitochondria, Atp11 is localized both in chloroplasts and mitochondria in Arabidopsis.

### Atp11 and Atp12 Interact With the β and α Subunits of mtATPase, Respectively

**FIGURE 4:**
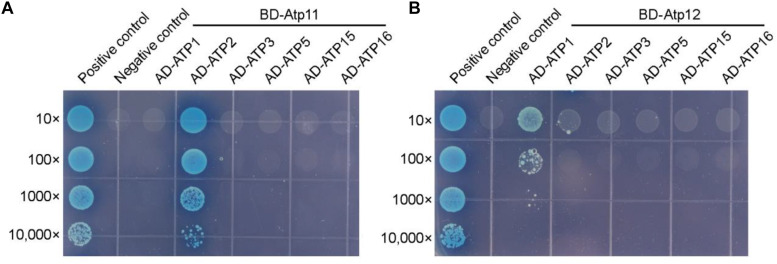
Arabidopsis Atp11 and Atp12 proteins interact with subunits of the mitochondrial ATP synthase. Yeast two-hybrid analysis of the interaction between Atp11 **(A)** and Atp12 **(B)** with subunits of the Arabidopsis mitochondrial ATP synthase. Mature Arabidopsis Atp11 and Atp12 proteins were fused to a vector as bait, while subunits of the Arabidopsis mitochondrial ATP synthase were fused to AD as prey. Cotransformation of pGBKT7-T with pGBKT7-53 or pGBKT7-Lam were used as positive and negative controls, respectively; 10- to 10,000-fold dilutions are shown.

Since the amino acid sequences of Atp11 and Atp12 are well conserved and both are localized in mitochondria (Figures 2, 3), it is reasonable to speculate that Atp11 and Atp12 of plants have similar roles as their presumed orthologs in yeast to facilitate the assembly of mtATPase in Arabidopsis. To verify this proposal, we determined interactions between Atp11 and Atp12 with subunits of the Arabidopsis mtATPase using the yeast two-hybrid system (Figure 4 and Supplementary Figure S1A). While mature Atp11 and Atp12 were fused to the bait vector, mtATPase F_1_ subunits were fused to the prey construct. As shown in Figure 4, Atp11 and Atp12 were found to specifically interact with the β (ATP2) and α (ATP1) subunits of the Arabidopsis mtATPase, respectively, indicating that Atp11 and Atp12 have a conserved function as their orthologs in *Saccharomyces cerevisiae*.

### Atp11 Also Interacts With the β Subunit of Chloroplast ATP Synthase

**FIGURE 5:**
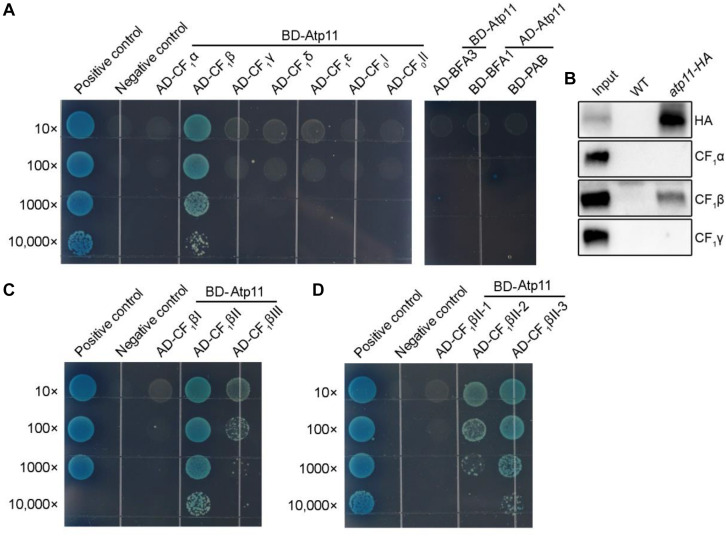
Atp11 specifically interacts with the CF_1_β subunit of Arabidopsis chloroplast ATP synthase. **(A)** Yeast two-hybrid analysis. Mature Atp11 was fused to a vector as bait, while the five CF_1_ subunits and two CF_*o*_ subunits of the chloroplast ATP synthase were fused to AD as prey. The interactions of chloroplast ATP synthase assembly factors (PAB, BFA1, and BFA3) and Atp11 were also analyzed. **(B)** Coimmunoprecipitation of Atp11 with CF_1_β in the *atp11-HA* transgenic line. Intact chloroplast isolated from wild type and *atp11-HA* plants were cross-linked with DSP and were then osmotically ruptured. Chloroplast stroma fraction was immunoprecipitated with anti-HA Affinity matrix. Immunoprecipitated proteins were detected by immunoblotting with HA, CF_1_β, CF_1_α, and CF_1_γ antibodies. **(C,D)** Yeast two-hybrid analysis of the sub-region of CF_1_β required for the interaction with Atp11. Cotransformations of pGBKT7-T with pGBKT7-53 or pGBKT7-Lam were used as positive and negative controls, respectively; 10- to 10,000-fold dilutions are shown.

Besides mitochondria, Atp11 was also localized in chloroplasts (Figure 3), suggesting that Atp11 may have a dual role in the two organelles in Arabidopsis. To determine whether Atp11 is involved in assembly of the cpATPase, subunits of the cpATPase were fused to the prey vector and were co-transformed with BD-Atp11 into yeast and then cultured on SD/-Leu/-Trp/-His/-Ade/X-a-gal medium to detect their interactions. Our results indicate that Atp11 specifically interacts with CF_1_β among the five CF_1_ subunits (CF_1_α to CF_1_ε) and two CF_*o*_ subunits (CF_*o*_I and CF_*o*_II) (Figure 5A and Supplementary Figure S1B), similar with its interaction with the β subunit of mtATPase (Figure 4A). To further investigate the interaction of Atp11 and CF_1_β *in vivo*, affinity chromatography using *Atp11-HA* plants was performed (Figure 5B). The results showed that CF_1_β, but not CF_1_α and CF_1_γ, are co-immunoprecipitated with Atp11-HA, confirming the specific interaction of Atp11 and CF_1_β in the chloroplast stoma *in vivo* (Figure 5B).

Therefore, to further map the binding site of Atp11 in the CF_1_β subunit, intact CF_1_β subunit was divided into three regions as previously described (Zhang et al., 2016, 2018). The results show that Atp11 strongly interacts with the CF_1_βII region and only weakly with the CF_1_βIII region (Figure 5C and Supplementary Figure S1C). The CF_1_βII region was further divided into three parts, CF_1_βII-1, CF_1_βII-2, and CF_1_βII-3 (Zhang et al., 2016, 2018). Y2H assays detected a weak interaction between Atp11 and CF_1_βII-2 and a strong interaction with CF_1_βII-3 (Figure 5D and Supplementary Figure S1C).

We previously showed that BFA3 and BFA1 are involved in the assembly of chloroplast ATP synthase and both of them interact with CF_1_β (Zhang et al., 2016, 2018). PAB is also an assembly factor required for assembly of chloroplast ATP synthase through its interaction with the CF_1_γ subunit (Mao et al., 2015). Our yeast two-hybrid analyses showed that Atp11p does not interact with BFA3, BFA1, and PAB (Figure 5A), although BFA3 and BFA1 also interact with CF_1_βII-2 as Atp11p in chloroplasts. Thus, Atp11 may promote the assembly of CF_1_β subunit via different mechanisms used by BFA1 and BFA3 in chloroplasts. This hypothesis is consistent with our previous finding that overexpression of Atp11 in *bfa3* could not complement the phenotype of *bfa3* (Zhang et al., 2016).

### Arabidopsis Atp11 Specifically Interacts With the βII (ATP2II) Sub-Region of the Mitochondrial ATP Synthase

**FIGURE 6:**
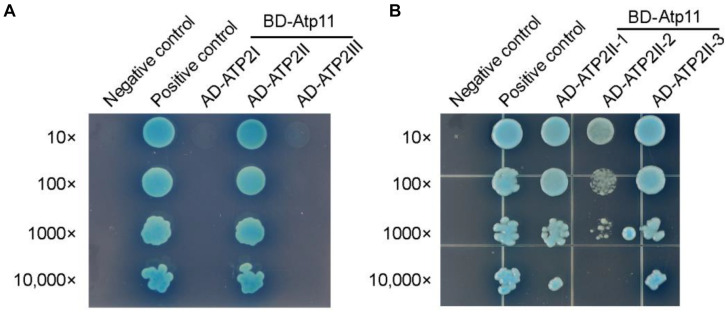
Atp11 specifically interacts with the ATP2II subregion of Arabidopsis mitochondrial ATP synthase. **(A,B)** Yeast two-hybrid analysis of the sub-region of ATP2 required for the interaction with Atp11. Mature Atp11 was fused to a vector as bait, while the six subregions were fused to AD as prey. Cotransformations of pGBKT7-T with pGBKT7-53 or pGBKT7-Lam were used as positive and negative controls, respectively; 10- to 10,000-fold dilutions are shown.

It has been shown that Atp11p from *Saccharomyces cerevisiae* binds to the nucleotide-binding domain of the β subunit (Wang and Ackerman, 2000). To detect the binding site of Atp11 in the mtATPase β (ATP2) subunit in Arabidopsis, intact mtATPase β subunit was divided into three regions as previously described (Zhang et al., 2016). Using the yeast two-hybrid system, we found that Atp11 specifically interacts with the ATP2II region (Figure 6A and Supplementary Figure S1D). The ATP2II region was further divided into three subregions and our results identified a weak interaction between Atp11 and ATP2II-2 and a strong interaction between Atp11 and the other two subregions (ATP2II-1 and ATP2II-3) (Figure 6B and Supplementary Figure S1D). These results differ slightly from those obtained with the cpATPase where Atp11 strongly interacts with the sub-regions of CF_1_βII-3 and slightly with CF_1_βII-2 and CF_1_βIII (Figures 5C,D). Moreover, Atp11 from *Saccharomyces cerevisiae* seems do not interact with the region of mtATPase β subunit corresponding to ATP2II-3 of the Arabidopsis mtATPase β subunit (Wang and Ackerman, 2000). These results suggest that the binding sites for Atp11 are slightly different in Arabidopsis and yeast mitochondria as well as in Arabidopsis chloroplasts.

## Discussion

Atp11p and Atp12p are members of two large chaperone families that were first discovered in studies of respiratory-deficient mutants of *Saccharomyces cerevisiae* (Ackerman and Tzagoloff, 1990). Subsequent analyses revealed that Atp11p and Atp12p are essential for assembly of mtATPase by binding to the β and α subunits, respectively (Wang and Ackerman, 2000; Wang et al., 2000; Ackerman and Tzagoloff, 2005), and their function is conserved during mtATPase assembly from yeast to humans (Wang et al., 2001; Ackerman, 2002). A blast search revealed that proteins structurally related to Atp11p and Atp12p are present in all photosynthetic eukaryotes including angiosperms, bryophytes, and the green alga *Chlamydomonas reinhardtii* (Figure 2) which contain two F-type ATP synthases (cpATP- and mtATP-synthase). However, it is unclear whether Atp11 and Atp12 function in the assembly of both organellar ATP synthases in photosynthetic eukaryotes.

In this work, we show that Atp12 is specifically localized in mitochondria but not in chloroplasts (Figure 3). Moreover, yeast two hybrid assays showed that Atp12 specifically interacts with mtATPase α subunit (Figure 4B), which is consistent with the localization and function of the Atp12p ortholog in yeast and *Homo sapiens*. These results imply that the function of Atp12 is conserved in mitochondrial ATPase assembly in higher plants. Disruption of Arabidopsis *ATP12* may lead to the loss of mtATPase, resulting in embryo lethality of *atp12* homozygous mutants (Figure 1). A comprehensive search of prokaryotic genomes revealed that Atp12p homologs are solely present in α-proteobacteria (Pícková et al., 2005). According to the endosymbiotic theory, mitochondria originated from α-proteobacteria and chloroplast from cyanobacteria (Osteryoung and Nunnari, 2003; Timmis et al., 2004). Although cyanobacteria contain F-type ATPases as chloroplasts, they do not have a homolog of Atp12. These facts suggest that Atp12p originated from a mitochondrial ancestor and is consistent with the appearance of mitochondria during the evolution of species. Why does assembly of mtATPase, but not cpATPase need Atp12p in Arabidopsis? As mentioned by Pícková et al. (2005), a peptide of nine amino acids is present within the binding site for Atp12p in the mtATPase α subunit that is missing in the chloroplast α subunit. Atp12 may be closely involved in the folding or assembly of this specific part of the α subunit. It is also possible that cyanobacteria and chloroplasts have evolved new chaperone protein(s) which fulfill a similar function as Atp12 in mitochondria.

**FIGURE 7:**
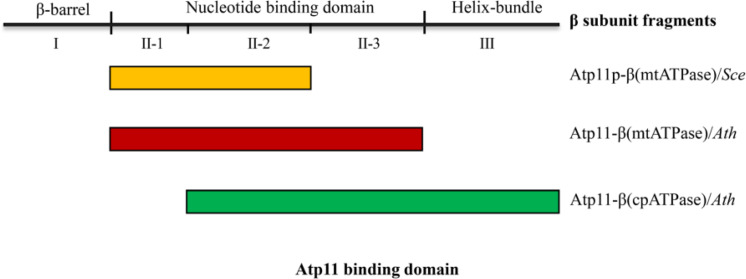
Interactions of Atp11 orthologs and β subunit from cpATPase/mtATP synthase in yeast and higher plants. This scheme summarizes the distinct patterns of the interactions between Atp11p from *Saccharomyces cerevisiae* (*Sce*) with mtATPase β subunit (yellow box) (Wang and Ackerman, 2000), Atp11 from *Arabidopsis thaliana* (*Ath*) with mtATPase β subunit (red box), and Atp11 from Arabidopsis with cpATPase β subunit (green box) (Figures 4–6).

Previous studies showed that no Atp11p homolog was detected in the α-proteobacterial genome and the origin of Atp11p is thought to be linked to the evolution of aerobic metabolism in eukaryotes (Pícková et al., 2005). Our results show that, in contrast to Atp12, Atp11 is localized both in chloroplasts and mitochondria in Arabidopsis and tobacco (Figure 3). It has been demonstrated that chloroplasts arose from a cyanobacterial ancestor acquired by a eukaryotic host in which mitochondria were already present (McFadden, 2001). Since cyanobacteria do not contain *Atp11* gene, chloroplast may have acquired Atp11p during the evolution of photosynthetic eukaryotes. Yeast two-hybrid analysis indicates that Atp11 interacts with both cp and mtATPase β subunits, consistent with the function of its orthologs (Figures 4–6). In addition, we found that the binding sites for Atp11 in the β subunit of the mt and cpATP synthases are slightly different (Figures 5–7). Moreover, these two binding sites are also different from the binding site for Atp11 in the mtATPase β subunit in *Saccharomyces cerevisiae* (Figure 7). These results imply that Atp11 fulfills a conserved function during ATP synthase assembly but the working mechanisms may have somehow diversely evolved.

Up to now, three cpATPase assembly factors PAB, BFA3, and BFA1 have been reported to transiently bind to subunits of the CF_1_ subcomplex in chloroplasts. However, we found that Atp11 does not interact with these three proteins (Figure 5A). These results indicate that Atp11, BFA1, and BFA3 may play different roles during the assembly of ATP synthase (Zhang et al., 2016, 2018, 2020). Moreover, BFA1 and BFA3 bind to CF_1_βII-2, a region in the catalytic site of CF_1_β subunit (Zhang et al., 2016, 2018). Atp11, however, was found to strongly interact with CF_1_βII-3 but only weakly with CF_1_βII-2 (Figure 5). Additionally, a weaker interaction of Atp11 with CF_1_βIII was also detected in yeast two-hybrid assays (Figure 5). These results indicate that Arabidopsis Atp11 plays a role in assembly of cpATPase but in a different way than BFA3 and BFA1. Further investigations are required to reveal the putative role of Atp11 in regulating cpATPase assembly in higher plants.

In conclusion, our results demonstrate that Arabidopsis Atp12 is specifically localized in mitochondria while Atp11 is present in both mitochondria and chloroplasts. As their orthologous proteins in yeast and humans, Arabidopsis Atp11 and Atp12 interact with the β and α subunits of ATPase, respectively, in their resident organelles, although the binding sites with their partners are slightly different. Thus, Atp11 and Atp12 are likely conserved chaperone family proteins essential for F-type ATPase assembly in all eukaryotes including the plant kingdom.

## Materials and Methods

### Plant Materials and Growth Conditions

The heterozygous *atp11-1* and *atp12-1* mutants were obtained from the NASC (SALK_018817 and GABI_516A01). All the mutants and transgenic *Arabidopsis thaliana* lines used in this study were in the Col-0 background. Plants were grown in soil in a growth chamber under controlled greenhouse conditions with a 16 h-light/8 h-dark photoperiod and at a light intensity of 50 μmol photons m^–2^ s^–1^ at 23°C. Six-week-old plants of *atp11-1* and *atp12-1* heterozygotes were used to analyze the embryo lethal phenotypes. To produce the transgenic lines, genomic DNA of *Atp11* and *Atp12* including promoter were cloned into the plant expression vector pCAMBIA1301 (Wang et al., 2020). In addition, the genomic DNA of *Atp11* was fused with the sequence encoding the HA tag and then cloned into pCAMBIA1301 to produce the *atp11-HA* plants. The resulting vectors were introduced into the heterozygous *atp11-1* and *atp12-1* mutants by the *Arabidopsis* floral-dip method with *Agrobacterium tumefaciens* (Clough and Bent, 1998). Transgenic lines were screened on Murashige and Skoog medium with hygromycin B and were further confirmed by PCR.

### Multiple Sequence Alignment

Sequences of Atp11 and Atp12 proteins in various species were obtained from GenBank^3^ or Phytozome^4^. Multiple sequence alignment of these proteins was generated with ClustalW2.

### Subcellular Localizations of Atp11 and Atp12

For subcellular localization of Atp11 and Atp12, full size Atp11 or Atp12 was subcloned into the pCAMBIA2300-P35S-GFP vector with GFP at the C terminus (Xing et al., 2019). For protoplasts transformation, the 35Spro:Atp11-GFP or 35Spro:Atp12-GFP constructs were transformed into protoplasts of *Arabidopsis* (Zhang et al., 2019). The mitochondrial marker MitoTracker Red CMXRos at a final concentration of 500 nM was incubated with the protoplasts for 15 min in the dark before imaging. The fluorescence of transiently transformed *Arabidopsis* protoplasts was observed with a Zeiss LSM 780 inverted microscope with a 488-nm laser. For MitoTracker Red, 561 nm excitation and 572–621 nm emission and for chlorophyll auto-fluorescence, 488 and 650–750 nm were used for excitation and emission, respectively. For double track imaging, GFP and chlorophyll auto-fluorescence were activated independently.

### Quantitative RT-PCR

Three-week-old wild-type and *atp11-HA* plants were used for qRT-PCR. Total RNA was extracted using TRIzol reagent (Invitrogen) and reverse transcribed with M-MLV reverse transcriptase (Promega). qRT-PCR was performed with ChamQ Universal SYBR qPCR Master Mix (Vazyme) and *Actin 2* gene was used as control (Qin et al., 2020).

### Isolation of Chloroplast Stromal Proteins and Thylakoid Membranes

Chloroplast stromal proteins and thylakoid membranes were isolated as previously described (Zhang et al., 2016, 2018). Leaves of 3-week-old transgenic lines (*atp11*-HA) were fully homogenized. Intact chloroplasts were carefully isolated in Medium II buffer (0.33 M sorbitol, 20 mM HEPES/KOH, pH 7.6) and then osmotically ruptured in Medium II buffer without sorbitol. Thylakoid membranes and stromal proteins were separated from each other by centrifugation (12,000 × *g* for 10 min at 4°C). A Protein Assay Kit (Bio-Rad) was used to determine the concentration of stromal proteins. Chlorophyll content was determined as described by Porra et al. (1989).

### Isolation of Mitochondria

Isolation of mitochondria was carried out as previous described (Sweetlove et al., 2007). Leaves from 3-week-old WT and *atp11-HA* plants were fully homogenized in isolation buffer containing 0.3 M sucrose, 5 mM tetrasodium pyrophosphate, 10 mM KH_2_PO_4_, pH 7.5, 2 mM EDTA, 1% PVP40, 1% BSA, 5 mM cysteine and 20 mM ascorbic acid and centrifuged at 5000 × *g* for 10 min. Crude mitochondria were collected form the resulting supernatant by centrifugation at 20,000 × *g* for 10 min and resuspended in the washing buffer (0.3 M sucrose, 1 mM EGTA, and 10 mM MOPS/KOH, pH 7.2). Crude mitochondria were further separated by centrifugation on a Percoll density gradient consisting of 18%, 25%, and 50% Percoll solution at 40,000 × *g* for 55 min. The 25–50% Percoll interface containing intact mitochondria was collected and diluted threefold with washing buffer. Mitochondria were collected by centrifugation at 20,000 × *g* for 10 min. The concentration of mitochondrial proteins was determined by a DC Protein Assay kit (Bio-Rad, 5000116).

### Immunoblot Analysis

For immunoblotting, thylakoid membranes were resuspended in sample buffer (50 mM Tris-HCl, pH 6.8, 5% SDS, 20% glycerol, 8 M urea, 5% 2-mercaptoethanol, 1% bromophenol blue) and incubated for 30 min at room temperature. Stromal and mitochondrial proteins were mixed with an equal volume of the sample buffer (2X). Protein samples were separated by SDS-urea-PAGE and transferred to nitrocellulose membranes. Immunoblot signals were measured by the chemiluminescence method with a LuminoGraph WSE-6100 (ATTO Technology).

### Antibody Production and Antibody Source

To express the mature Atp11 recombinant protein, the pET28a expression vector harboring the cDNA sequence encoding the mature Atp11 protein (amino acids 87–248) was constructed. Recombinant protein was induced with 1mM IPTG in *E. coli* BL21 (DE3) strain and purified using Ni-NTA agarose under denaturing conditions. The polyclonal Atp11 antibodies were produced in rabbits (PhytoAB, United States) and were used at a 1:1000 dilution. Antibodies against RbcL (PhytoAB, PHY0346), D1 (PhytoAB, PHY0057), HA tag (Abmart), ATP3 (PhytoAB, PHY0595S), RPE (D-RIBULOSE-5-PHOSPHATE-3-EPIMERASE, PhytoAB, PHY0616), CF_1_α (PhytoAB, PHY0311), CF_1_β (PhytoAB, PHY0312), CF_1_γ (PhytoAB, PHY0313) were purchased from a commercial supplier and used at a 1:1000 dilution.

### Immunoprecipitation

For immunoprecipitation, preimmune serum and the antibody against Atp11 were purified with Protein A Sefinose (TM) Resin (Sangon Biotech) and then coupled to CNBr-activated agarose (Sangon Biotech). Chloroplast stroma isolated from WT and *atp11-HA* plants was denatured with 2% SDS for 10 min at 25°C and then 30 s at 70°C. After centrifugation at 15,000 × *g* for 10 min, the supernatant was transferred to a new tube and diluted 10-fold with IP buffer (50 mM Tris-HCl, pH 8.0, 100 mM NaCl, 1 mM EDTA, 0.1% Igepal CA-630). A total of 100 μl antibody-coupled CNBr-activated agarose was added and incubated overnight at 4°C. The beads were washed seven times with IP buffer and one time with washing buffer (50 mM Tris-HCl, pH 7.5). Bound protein was eluted with 50 μl of 1.5X Laemmli buffer (90 mM Tris-HCl, pH 6.8, 3% SDS, 10% glycerol, and 0.01% bromophenol blue) and 10 μl of sample was used for immunoblot analysis with antibodies against Atp11.

### Yeast Two-Hybrid Assays

Yeast two-hybrid assays were conducted following the Matchmaker Gold Yeast Two-hybrid System (Clontech). The sequences encoding mature Atp11 and Atp12 were fused to the bait vector pGBKT7, respectively. The coding sequences of subunits of the chloroplast and mtATPase were cloned into the prey vector pGADT7. Prey and bait plasmids were transformed into the yeast strain Y2H Gold by the small-scale lithium acetate method. Positive colonies were grown on SD/-Trp-Leu medium for 3 days to confirm the transformation with both bait and prey plasmids and cultured on SD/-Leu/-Trp/-His/-ade/X-a-Gal medium to detect protein–protein interactions.

### Other Methods

Affinity chromatography of Atp11-HA associated protein was performed as previous described (Zhang et al., 2016, 2018). Plastid-targeting signals (cTP) of Atp11 and Atp12 proteins were predicted by ChloroP 1.1 Server (see text footnote 1) and TargetP 2.0 Server^5^.

### Accession Numbers

Sequence data from this article can be found in the National Center for Biotechnology Information or Phytozome databases database under the following accession numbers: AthATP11, *Arabidopsis thaliana*, AT2G34050; GmaATP11, *Glycine max*, XP_003539932.1; OsaATP11, *Oryza sativa*, XP_015623575.1; ZmaATP11, *Zea mays*, NP_001150475.2; SmoATP11, *Selaginella moellendorffii*, XP_002980989.1; PptATP11, *Phys comitrella patens*, Pp3c14_19140V3.1; CreATP11, *Chlamydomo nas reinhardtii*, Cre10.g437050.t1.2. AtATP12, *Arabidopsis thaliana*, AT5G40660; GmaATP12, *Glycine max*, XP_003547684.1; OsaATP12, *Oryza sativa*, XP_015645080.1; ZmaATP12, *Zea mays*, NP_001148797.2; SmoATP12, *Selaginella moellendorffii*, XP_002980146.1; PptATP12, *Physcomitrella patens*, Pp3c2_19250V3.2; CreATP12, *Chlamydomonas reinhardtii*, Cre17.g726250.t1.2.

## Data Availability Statement

The datasets generated for this study are available on request to the corresponding author.

## Author Contributions

ZD, CL, and LP conceived the study and designed experiments. ZD, KL, LZ, LC, and LL performed experiments. All authors analyzed the data. ZD and LZ produced the figures. LZ, J-DR, and LP wrote the manuscript. LP supervised the whole study.

## Conflict of Interest

The authors declare that the research was conducted in the absence of any commercial or financial relationships that could be construed as a potential conflict of interest.

## Footnotes

1 http://www.cbs.dtu.dk/services/ChloroP

2 http://www.cbs.dtu.dk/severces/TMHMM-2.0/

3 http://www.ncbi.nlm.nih.gov/

4 http://www.phytozome.net/

5 http://www.cbs.dtu.dk/services/TargetP/

## Supplementary Material

Supplementary Figure 1 Growth of indicated yeast strains on Sd/-Leu/Trp plates. As growth and transformation controls, yeast co-transformed with various vectors as in Figures 4–6 were cultured on Sd/-Leu/Trp. 10- to 10,000-fold dilutions are shown.

Supplementary Table 1 Ratio of normal to aborted embryos in the progeny of Wt, *atp11*^–/+^ and *atp12*^–/+^ mutants.

Supplementary Table 2 Segregation in the progeny of *atp11*^–/+^ and *atp12*^–/+^ mutants.

Supplementary Table 3 Primers used in this work.
