# Supplementary figures and images for: F-Type ATP Synthase Assembly Factors Atp11 and Atp12 in Arabidopsis
Source: Front Plant Sci. 2020 Oct 19;11:522753. doi: 10.3389/fpls.2020.522753 (PMC7607909; doi:10.3389/fpls.2020.522753)

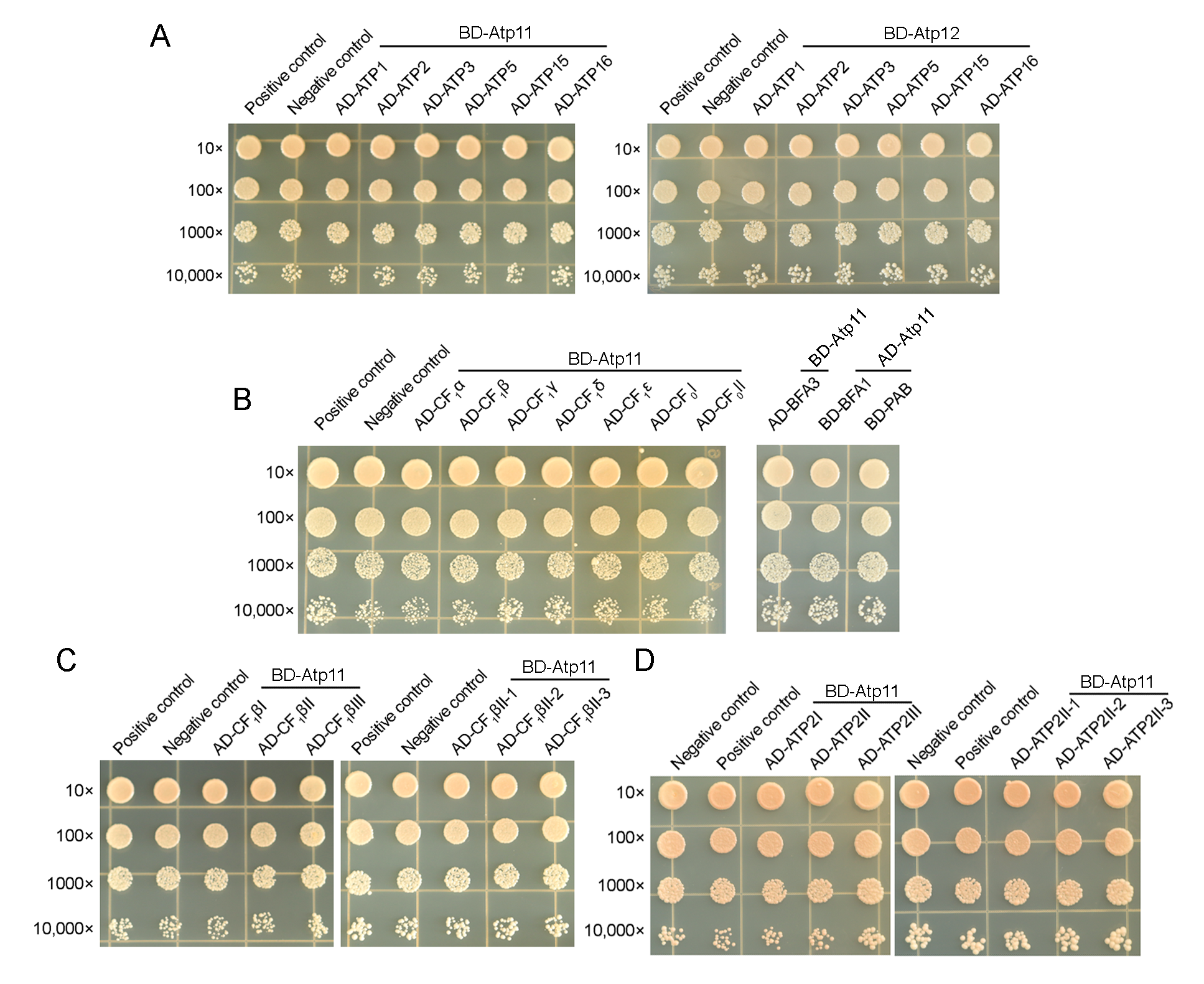

Supplement: Supplementary Figure 1 — Growth of indicated yeast strains on Sd/-Leu/Trp plates. As growth and transformation controls, yeast co-transformed with various vectors as in Figures 4–6 were cultured on Sd/-Leu/Trp. 10- to 10,000-fold dilutions are shown. [file Image_1.tif]
