# Supplementary material for: F-Type ATP Synthase Assembly Factors Atp11 and Atp12 in Arabidopsis
Source: Front Plant Sci. 2020 Oct 19;11:522753. doi: 10.3389/fpls.2020.522753 (PMC7607909; doi:10.3389/fpls.2020.522753)
Supplement: Supplementary Table 1 — Ratio of normal to aborted embryos in the progeny of Wt, atp11–/+ and atp12–/+ mutants. [file Table_1.docx]

**Supplemental Table 1**. Ratio of normal to aborted embryos in the progeny of WT, *atp11^-/+^* and *atp12^-/+^* mutants.

|  | Total embryos | Normal embryos | Aborted embryos | Ratio  (normal/aborted embryos) |
| --- | --- | --- | --- | --- |
| WT | 386 | 380 | 6 | 63.33:1 |
| *atp11^-/+^* | 374 | 282 | 92 | 3.07:1 |
| *atp12^-/+^* | 363 | 275 | 88 | 3.13:1 |
