# Supplementary material for: F-Type ATP Synthase Assembly Factors Atp11 and Atp12 in Arabidopsis
Source: Front Plant Sci. 2020 Oct 19;11:522753. doi: 10.3389/fpls.2020.522753 (PMC7607909; doi:10.3389/fpls.2020.522753)
Supplement: Supplementary Table 2 — Segregation in the progeny of atp11–/+ and atp12–/+ mutants. [file Table_2.docx]

**Supplemental Table 2**. Segregation in the progeny of *atp11^-/+^* and *atp12^-/+^* mutants.

|  | Total | WT | Heterozygous | Homozygous | Ratio  (WT/Heterozygous) |
| --- | --- | --- | --- | --- | --- |
| *atp11^-/+^* | 95 | 34 | 61 | 0 | 1:1.79 |
| *atp12^-/+^* | 84 | 31 | 53 | 0 | 1:1.71 |
