# Supplementary material for: F-Type ATP Synthase Assembly Factors Atp11 and Atp12 in Arabidopsis
Source: Front Plant Sci. 2020 Oct 19;11:522753. doi: 10.3389/fpls.2020.522753 (PMC7607909; doi:10.3389/fpls.2020.522753)
Supplement: Supplementary Table 3 — Primers used in this work. [file Table_3.docx]

**Supplemental Table 3.** Primers Used in This Work.

| **Primer names** | **Sequences 5’ to 3’** |
| --- | --- |
| **Primers used for mapping and detection of T-DNA insertion sites** | |
| 11HAF  11HAR  12HAF  12HAR  LBa1  TLB2 | ATGGTTACATAACAATGCTTGCTCC  GGTGTTCTTTTGGAAGAAGAAGACT  TCAACTTAAAGCTGAGAGCTTACCG  TTGCCTACAACAAGCAATAAAACTC TGGTTCACGTAGTGGGCCATTCG  TGCTTCGCCTATAAATACGACG |
| **Primers used for qPCR** | |
| ATP11-qPCR-F | TTTGCTCAAGTTGAAGCGCC |
| ATP11-qPCR-R | TAGTCAGGTAAGGAGCCGCT |
| Actin2-qPCR-F | GTTGGTGATGAAGCACAATCCAAG |
| Actin2-qPCR-R | CTGGAACAAGACTTCTGGGCATCT |
| **Primers used for antiserum production** | |
| ATP11-Ab-F | CGGGATCCGCCAAGACTAAATCCCCTGAAG |
| ATP11-Ab-R | CCCAAGCTTCAGAAGAGGCATATCCAGAGC |
| **Primers used for complementation and subcellular localization vector construction** | |
| ATP11-com-F  ATP11-com-R  ATP12-com-F  ATP12-com-R  ATP11-HA-F  ATP11-HA-R  ATP11-GFP-F  ATP11-GFP-R  ATP12-GFP-F  ATP12-GFP-R | CGGAATTCCAATATCGACCCATCACAACC CGGGATCCTCACAGAAGAGGCATATCCA  CCGAGCTCCCATAAGTGTCAAAGTAC  CGGGATCCTCAGTTTTCACGGGATAG  CGGAATTCCAATATCGACCCATCACAACC CGGGATCCCAGAAGAGGCATATCCAGAG  CAGGTACCATGAGAAGAATCGTCGGTTCAA  ATGGATCCCAGAAGAGGCATATCCAGAGCT  ATGGTACCATGGCGGCGATGTTAATCGGAA  GCTCTAGAGTTTTCACGGGATAGAGCAAGA |
| **Primers used for yeast two-hybrid vector construction** | |
| ATP11-BD-F  ATP11-BD-R  ATP11-AD-F  ATP11-AD-R  ATP12-BD-F  ATP12-BD-R  CF_1_α-AD-F  CF_1_α-AD-R  CF_1_β-AD-F  CF_1_β-AD-R  CF_1_γ-AD-F  CF_1_γ-AD-R  CF_1_δ-AD-F  CF_1_δ-AD-R  CF_1_ε-AD-F  CF_1_ε-AD-R  CFoI-AD-F  CFoI-AD-R  CFoII-AD-F  CFoII-AD-R  CF_1_βI-AD-F  CF_1_βI-AD-R  CF_1_βII-AD-F  CF_1_βII-AD-R  CF_1_βIII-AD-F  CF_1_βIII-AD-R  CF_1_βII-1-AD-F  CF_1_βII-1-AD-R  CF_1_βII-2-AD-F  CF_1_βII-2-AD-R  CF_1_βII-3-AD-F  CF_1_βII-3-AD-R  ATP1-AD-F  ATP1-AD-R  ATP2-AD-F  ATP2-AD-R  ATP2I-AD-F  ATP2I-AD-R  ATP2II-AD-F  ATP2II-AD-R  ATP2II-1-AD-F  ATP2II-1-AD-R  ATP2II-2-AD-F  ATP2II-2-AD-R  ATP2II-3-AD-F  ATP2II-3-AD-R  ATP2III-AD-F  ATP2III-AD-R  ATP3-AD-F  ATP3-AD-R  ATP5-AD-F  ATP5-AD-R  ATP15-AD-F  ATP15-AD-R  ATP16-AD-F  ATP16-AD-R  BFA3-AD-F  BFA3-AD-R  BFA1-BD-F  BFA1-BD-R  PAB-BD-F  PAB-BD-R | AATGAATTCAAATGGGCTTCGCTTGG TGAGTCGACCAGAAGAGGCATATCC  ACTCATATGAAATGGGCTTCGCTTGG  GCGCTCGAGCAGAAGAGGCATATCC  TATGAATTCCAACCCGATTCCGATACTCAG  TAAGTCGACGTTTTCACGGGATAGAGCAAG  ACTCATATGATGGTAACCATTAGAGCCGAC  AGCGTCGACTACTTTCTCCTGAAGTAGG  CGCGAATTCATGAGAACAAATCCTAC  GCGCTCGAGTTTCTTCAATTTACTC  CCACATATGGCGTCTCTTCGTGAG  ATCCTCGAGAACCTGTGCATTAGCTC  ATACATATGGCCACCGCAGCATCAAG  GCGGAATTCAGTAGCTAATTGAATCTCACC  CGACATATGATGACCTTAAATCTTTG  TATGAATTCAATCGTATTGAGAGCCTCG  GCGGGATCCATGATTTATTAGATAACCG  AGTCTCGAGATCAGTTATTTCTTTC  TATGAATTCCCGCTTGGTAACTTCATGG  GATGGATCCAGAAGGAAGAACCTTCTTGACAAT  CGCGAATTCATGAGAACAAATCCTAC  ACTCTCGAGATTTCCCATATCAACCAC  ATGGAATTCCCTCTAAGTGTTCCAG  AACCTCGAGAGGTTGTAGCATAGTTG  CTAGAATTCCGAATCGTTGGCGAG  GCGCTCGAGTTTCTTCAATTTACTC  ATGGAATTCCCTCTAAGTGTTCCAG  AATCTCGAGCCCACGACGATAAG  CGTGAATTCGGAAAAATTGGACTATTCGG  GCGCTCGAGGATATTGTCGATAAATAG  GATGAATTCTTCCGTTTCGTACAAGC  AACCTCGAGAGGTTGTAGCATAGTTG  GCCGAATTCATGGAATTATCTCCTAGAG  GCGCTCGAGAATTAAAGCTAAAGCTC  TATGAATTCTCAGCTGCGCCATCTTC  GCTGAGCTCAGCTGCTGACTCTTTAG  TATGAATTCTCAGCTGCGCCATCTTC  ACTGAGCTCAGGTACAGTGATTGGAG  CATGAATTCGTCGGAAGGGCTACTCT  ACTGAGCTCGTGAGGTGACAGCATACG  CATGAATTCGTCGGAAGGGCTACTCT  ATTGAGCTCTCCTCCTCTTTGGTAAG  AATGAATTCAAGATTGGTCTCTTTGGC  GCCGAGCTCAATGTTGTCAATGAAAAG  TATGAATTCTTCCGTTTCACTCAGGCC  ACTGAGCTCGTGAGGTGACAGCATACG  TTTGAATTCATTCTGGGAGAGGAGCAT  TCTGAGCTCAGCTGCTGACTCTTTAGC  ATGGAATTCGCTTCCAAGCTTAGAG  ATACTCGAGTTTAGCAGCTTCAAGAGC  ACTATCGATGCTCAAACAACCGCTAATG  AGACTCGAGGAGGTTGTTGAAGTCAAC  GTCGAATTCAATATCGTGAGGAACTG  TTACTCGAGAACTTCAGGTGTGTCTG  ATCGAATTCGCATCAACAGGACAAATG  ATTCTCGAGGCCCGAGAGAGCTGCGTT  TCTGAATTCTCTGGACTCCATTTGAACCG  GATGGATCCCATAATTTCATTAGCCCTGGAC  TTAAGATCTCCATGACGGTTCAAGAAG  AGACTGCAGCATTTTTGTCTCAGTAAA  TATCATATGAAGATGTTGCTGCCAC  CGAGTCGACATCTGTGTACAAAACG |
